# Social Enrichment Improves Affective State and Foraging Behavior Compared to Physical Enrichment, While Maintaining Growth Performance in Broiler Chickens

**DOI:** 10.3390/ani14223186

**Published:** 2024-11-06

**Authors:** Sofía Herrera-Alcaíno, Daniela Luna, Jorge González-Pavez, Paloma Cordero, Sergio A. Guzmán-Pino

**Affiliations:** 1 Programa de Doctorado en Ciencias Silvoagropecuarias y Veterinarias, Campus Sur, Universidad de Chile, Santiago 8820808, Chile; sofia.herrera.a@ug.uchile.cl (S.H.-A.); paloma.cordero@veterinaria.uchile.cl (P.C.); 2 Departamento de Fomento de la Producción Animal, Facultad de Ciencias Veterinarias y Pecuarias, Universidad de Chile, Santiago 8820808, Chile; daniela.luna.f@uchile.cl (D.L.); jorgegonzalezp.vet@gmail.com (J.G.-P.)

**Keywords:** broiler chickens, poultry behavior, animal welfare, human-animal interaction, attention bias, tonic immobility, affective state

## Abstract

Environmental enrichment is key for improving the broiler welfare in intensive production systems. This study lasted for 43 days and evaluated the effects of physical and social enrichment on broiler welfare. A total of 96 one-day-old male broilers were divided into two treatments: control treatment, provided with physical enrichment (*n* = 48) including perches, platforms, and straw bales; and social treatment (*n* = 48), involving regular positive interactions with a human. The behavioral repertoire, footpad health, taste preferences for sweet and umami compounds, affective states (through tonic immobility and attention bias tests), and growth performance were evaluated. No effects were found for feeding, drinking, locomotion, lying, or standing behaviors, although differences were observed at specific time points: the Social group had more feeding time on day 21 (*p* = 0.046), while physical treatment showed reductions on days 28 and 35 (*p* < 0.001). The Social group showed increased drinking on day 42 (*p* < 0.001), reduced locomotion (*p* = 0.022), and increased lying (*p* = 0.046) on day 42. Foraging was higher in the Social group (*p* = 0.027), but preening showed no differences. Footpad lesions showed no significant differences. The Control group preferred sucrose 100 mM (*p* = 0.022), but no preference for MSG 300 mM was observed. With regard to tonic immobility, the Control group showed a tendency towards larger durations (*p* = 0.078), indicating a potential increase in fearfulness. In the attention bias test, although the freezing behavior increased, the latency to begin feeding was shorter in the Social group (*p* < 0.001), suggesting an improved affective state. Lastly, no significant differences were observed for any of the growth parameters, including average daily feed intake, average daily gain, feed conversion ratio, or final body weight, indicating that the Social group maintained productivity. This approach provides a practical solution for improving the quality of life for broilers in intensive systems.

## Simple Summary

Broiler chickens in intensive production systems are often kept in environments with low sensory input, which can negatively impact their welfare. This study investigated the effects of physical and social enrichments. We examined how these strategies influenced the chickens’ behavior, physiological responses, overall welfare, and growth parameters. Both types of enrichment improved their welfare, but social enrichment was particularly effective in encouraging foraging behavior and seemed to promote a more positive affective state. It is noteworthy that these welfare improvements maintained both growth and productivity, as no differences were observed in the productive parameters. These results suggest that incorporating social interactions into poultry farming practices can enhance animal welfare and maintain the production efficiency, offering a practical approach to improving the conditions in intensive farming systems.

## 1. Introduction

High stocking densities in intensive broiler chicken production systems, up to 42 kg/m^2^, often result in issues such as lameness and contact dermatitis [1,2]. Rapid growth, low activity, and wet litter exacerbate these conditions, negatively affecting the health and welfare of the birds [3,4,5]. Environmental enrichment has been proposed to improve broiler welfare by introducing elements that encourage natural behaviors and reduce stress [6]. Newberry [7] defines environmental enrichment as a modification of the environment of captive animals, thereby increasing the animal’s behavioral possibilities and leading to improvements in biological function. Specifically, environmental enrichment is designed to (1) increase the occurrence and range of species-specific behaviors, (2) prevent or reduce abnormal behaviors, (3) enhance the positive utilization of the environment, and (4) improve the animal’s ability to handle behavioral and physiological challenges [6]. Importantly, enrichment must be biologically relevant to the species and its natural behaviors to be effective.

Physical enrichment involves modifications to the broiler’s environment through the introduction of elements like perches, barriers, and platforms [8]. These features increase movement, encourage exploration [9,10], and support leg health by motivating locomotor activity, which has been shown to reduce the incidence of leg disorders and improve the overall welfare [6]. Social enrichment includes both direct and indirect interactions with conspecifics or humans [8]. These enrichment strategies, as outlined by Bloomsmith et al. [11], are designed to cater to the different needs of animals, promoting their overall welfare in captivity.

Recent studies have shown that increased locomotion positively impacts leg skeletal development in broilers [12,13]. The use of physical enrichment, such as barriers, perches, ramps, and straw bales, improves leg health by motivating movement [14,15]. Early exposure to enrichment allows birds to express their natural behaviors, significantly enhancing their welfare [15,16]. Straw bales and platforms have been found to increase exploratory and locomotor activity, reduce pododermatitis, and lower fear responses [17,18,19,20]. Additionally, Mocz et al. [21] reported that elevated platforms and straw bales help reduce pododermatitis and walking difficulties, even at high stocking densities. Despite the growing body of research on physical enrichment for broilers, such as perches and straw bales, there is comparatively little research focused on social enrichment, particularly on human–animal interactions [22,23]. While social enrichment in other livestock species has been shown to improve their welfare through positive interactions with humans [24], the studies on broiler chickens are limited. Al-aqil et al. [22] found that chickens exposed to positive human interaction showed reduced levels of physiological stress and fear-related behaviors compared to those that did not receive positive human contact, highlighting the importance of human handling in reducing transportation stress.

Beyond the benefits of physical and social enrichment of behavior and health, taste preference assessments have been used to explore how chickens respond to specific compounds, such as sweet and umami tastes. Forbes [25] demonstrated that the selection of taste compounds is associated with increased comfort and positive affective states in animals. This suggests that the act of choice itself serves as a strategy to enhance emotional welfare. In non-enriched environments, broilers often prefer sweet and umami compounds, likely using these preferences to reduce stress and improve their overall welfare [25]. However, as observed in previous research by Cordero et al. [26], these taste preferences are more evident in chickens raised without enrichment, likely as a compensatory mechanism to improve their comfort in less favorable conditions. In non-enriched environments, broilers often show a preference for these compounds, likely as a means to reduce their stress and improve their welfare [25]. Previous research by Cordero et al. [26] showed these preferences in chickens raised in non-enriched settings.

In addition to promoting physical and mental health, enrichment strategies can significantly influence the affective state of animals [27,28]. Affective states refer to the emotional conditions of the animals, which can range from short-term responses like fear to long-term emotional states such as anxiety [27]. Assessing affective states is crucial for understanding the emotional well-being of animals. In recent years, methods such as the attention bias test have been used to evaluate the anxiety and fear responses in poultry, providing insights into how enrichment can alleviate negative emotional states [27].

Cransberg et al. [23] found that high levels of fear towards humans can significantly limit productivity, as fear responses are associated with increased stress and reduced growth performance. For instance, in broiler chickens, high levels of fear can interfere with normal feeding and foraging behaviors, ultimately affecting weight gain and feed conversion ratios. However, further research is needed to fully understand the effects of human–animal relationships on the affective state of broiler chickens, in addition to other welfare indicators such as foot health and growth performance.

In this study, we adopted the EU standards as a reference [2] and used physical enrichment as the control treatment to reflect the standard practices in modern poultry production. Including an unenriched control group was not deemed appropriate for ethical and scientific reasons, as previous research consistently demonstrates that enrichment reduces stress and promotes natural behaviors. By comparing social enrichment with physical enrichment, we explored whether positive human-animal interactions could deliver additional welfare benefits beyond the current industry standard.

This study hypothesized that broiler chickens exposed to regular positive human interactions (social enrichment) will demonstrate an increased expression of exploratory behaviors, such as locomotion and foraging activity, reduced footpad lesions, a preference for sweet and umami taste compounds, a more positive affective state, and maintained growth performance compared to birds that receive only physical enrichment without any human interaction. The study aimed to assess the influence of physical and social environmental enrichment on the behavioral repertoire, footpad health, sweet and umami preferences, the affective state, and growth performance of broiler chickens.

## 2. Materials and Methods

All the experimental procedures were conducted at the Experimental Unit for Poultry Nutrition and Production of the Faculty of Veterinary and Animal Sciences (FAVET) at the University of Chile (UCH).

### 2.1. Animals, Housing, and Management

A total of 96 one-day-old male broiler chickens (Ross 308) were selected to be used in the experiment. The chickens were obtained from a commercial poultry farm in the Metropolitan Region of Chile and distributed in a facility with 16-floor pens (1.00 m in width, 2.00 m in length, and 1.70 m in height). The stocking density was calculated as 9 kg/m^2^. The facility had natural ventilation and temperature control via gas heaters with thermostats. Each pen had wood shavings bedding which was not changed and remained the same throughout the entire experimental period. The feeding program consisted of commercial starter and grower diets formulated to meet the nutritional requirements established by the NRC [29] and the guidelines set by the breeder [30]. The starter diet provided 3069 kcal/kg of metabolizable energy, 19.94% crude protein, and 5.84% total minerals, and was offered from 1 to 23 days. The grower diet, provided from 24 to 42 days, contained 3142 kcal/kg of metabolizable energy, 17.00% crude protein, and 5.19% total minerals. Each diet was offered ad libitum to the chickens from 1 to 23 days and 24 to 42 days, respectively. Water was also provided ad libitum until the onset of the preference tests. The dark and light cycles followed the breeder’s guidelines [31] with 23 h of light and 1 h of darkness from 0 to 7 days, 19 h of light and 5 h of darkness from 8 to 40 days, and returning to 23 h of light and 1 h of darkness from 41 to 43 days. The rate of mortality recorded was 3%. At the end of the study, the animals were euthanized using cervical dislocation, following the recommendations and guidelines of the AVMA (American Veterinary Medical Association) to ensure a humane and ethical procedure.

### 2.2. Experimental Design

**Figure 1:**
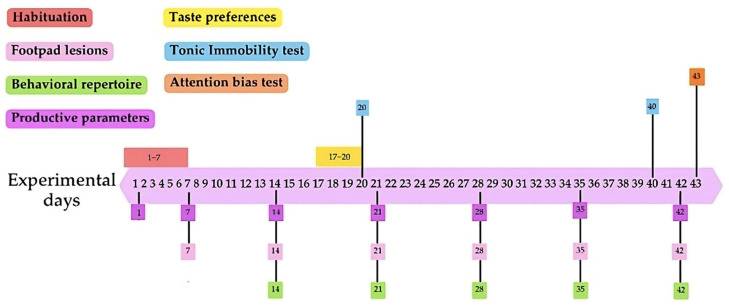
Timeline of specific measurements. Each measurement or test is represented by a different color: habituation period (red), footpad lesions (pink), behavioral repertoire (green), productive parameters (purple), taste preferences (yellow), tonic immobility test (blue), and attention bias test (orange). The boxes indicate the day or range of days on which each measurement or test was conducted during the experimental period.

Upon arrival, chicks were weighed for subsequent homogeneous distribution, with an average individual chick weight of 40.96 g (±1.02 g). Six birds were allocated to each of the 16 pens, with 8 pens per treatment, resulting in 48 chickens per treatment, which were subjected to an initial 7-day habituation phase to the environmental conditions of the poultry unit (Figure 1). The experimental treatments began on day 7, following the habituation phase, and continued until the end of the 43-day experimental period.

**Figure 2:**
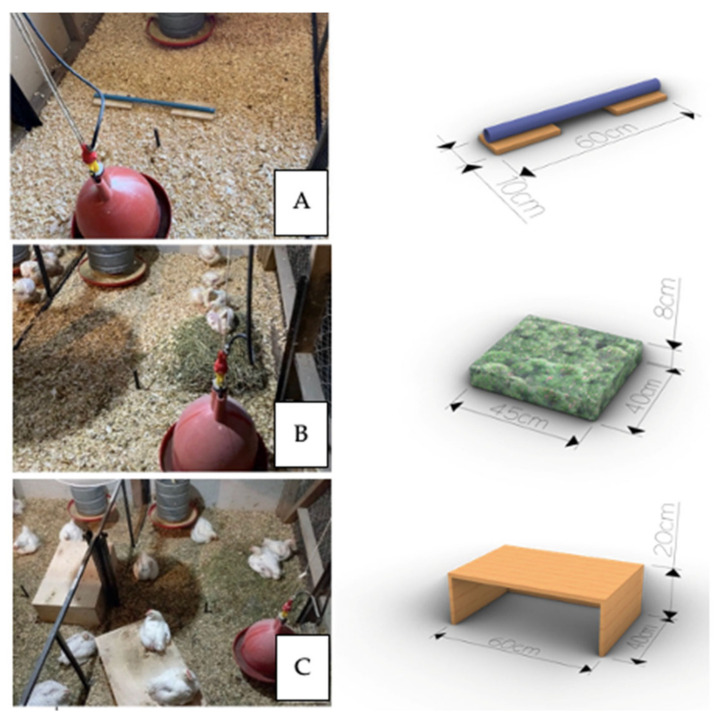
Photographic overview of the pen in Control treatment with enrichment references. (**A**) Circular PVC perches, (**B**) straw bale, and (**C**) wood platform.

The animals were assigned to one of the following two treatments: (1) The physical enrichment (Control) was implemented in 8 pens, each with a traditional bell drinker (diameter: 35 cm, height: 35 cm, plastic) and a circular feeder (diameter: 39 cm, height: 31 cm, metal and plastic). Water was provided through an automated system, requiring no operator intervention. Operators briefly entered the pens to refill the feeders, spending less than 20 s without interacting with the birds. The pens were enriched every two weeks with different physical elements to maintain their novelty and stimulate the birds’ natural behavior (Figure 2). In the second week (following habituation), circular PVC perches 60 cm long (with a diameter of 2 cm and a height of 1.5 cm) with wooden bases at both ends measuring 20 cm × 10 cm were installed (Figure 2A). In the fourth week, a straw bale measuring 45 cm long (L), 40 cm wide (W), and 8 cm high (H) was added and placed in a corner of the pen, providing enough space for the chickens to interact with the straw and perform natural behaviors such as exploration and pecking (Figure 2B). In the sixth week, a platform made of wood and nails, measuring 60 × 40 × 20 LWH cm was introduced (Figure 2C).

**Figure 3:**
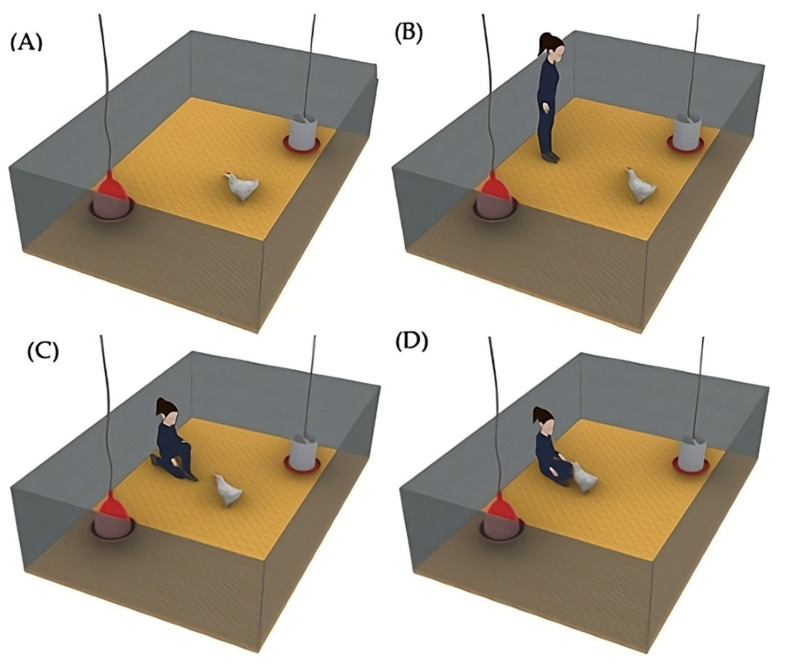
Referential image of each phase of the human–animal interaction: (**A**) caretaker entering the pen; (**B**) brief pause to observe; (**C**) caretaker seated, allowing bird approach; (**D**) gentle interaction with birds through stroking.

(2) The social enrichment (Social) was implemented in 8 pens, which only had a traditional drinker and feeder. During the experiment, the birds received regular human contact from the same person from Monday to Friday (Figure 3), in addition to routine management tasks, ensuring consistency in the interaction. On alternating days, the positive handling started in either increasing or decreasing order of pen numbers; for example, on one day, the interaction began with Pen 1 of the social treatment and proceeded sequentially to Pen 8, while on the next day, the interaction started with Pen 8 and proceeded in reverse order to Pen 1. Each day, the caretaker slowly entered the pen at 10 a.m. (Figure 3A) and, after waiting 5 s inside the pen (Figure 3B), calmly sat on the floor. The birds approached voluntarily (Figure 3C), and the caretaker performed gentle strokes using the fingertips (Figure 3D), primarily on the head and back. Each interaction session lasted 15 min. At the end, the female caretaker slowly stood up and exited the pen. The caretaker’s attire always consisted of dark green coveralls and black boots, ensuring a constant appearance for the birds. The person responsible for the routine cleaning tasks was different from the individual performing the gentle handling interactions.

Additionally, the pens were covered between treatments to avoid visual interference between them. Specifically, they were covered with Raschel mesh to prevent the chickens from seeing one another across the different treatment groups. This measure was implemented to minimize the potential biases, including those related to social learning, that could arise from visual contact between the Control and Social, ensuring that the observed outcomes were not influenced by the birds’ awareness of the presence of other treatment [32].

### 2.3. Behavioral Analysis

To evaluate the behavioral repertoire of broiler chickens, the birds’ behavior was recorded using IP WIFI 2MP cameras (Ezviz, SENKO INGENIERIA SPA, Santiago, Chile). The cameras were positioned at a height of 1.70 m above the pens. The cameras were securely mounted above the fencing, attached to the top of the pen structure, ensuring a stable and unobstructed view of the two pens simultaneously. The cameras were connected and monitored via Wi-Fi on a computer using software from the same brand. The recordings were stored on a 256 GB external hard drive for the subsequent analysis using BORIS’s behavioral coding software 7.13.9 [33]. One 5 min recording per pen was registered weekly after the habituation week on days 14, 21, 28, 35, and 42 before the weighing management (Figure 1). The records were made in the morning at 10 am. Videos were analyzed using an adapted ethogram from Sanchez-Casanova et al. [34], detailing the frequency and duration of the different behaviors observed in each treatment (Table 1). The analysis focused on tracking one bird at a time to capture all the observed behaviors accurately. Once the behavior of one bird was fully analyzed, the process continued with the next bird, ensuring comprehensive individual assessments for all the animals. No physical markers were used, relying entirely on the visual identification and tracking of each bird in the recordings.

### 2.4. Footpad Lesions

The incidence of footpad lesions was evaluated in all the broiler chickens from the Control and Social treatments. Throughout the 43-day experimental cycle, an observer conducted weekly visual assessments (every 7 days, excluding the first week of habituation) of each bird’s legs and extremities. These assessments were performed on days 7, 14, 21, 28, 35, and 42 during the weighing management (Figure 1). While handling the birds, we inspected their feet for any type of lesion, redness, or inflammation, following the Welfare Quality [35] guidelines (Table 2).

### 2.5. Sweet and Umami Taste Preference Analysis

One preference test was conducted between days 17 and 20 of the experimental period. The chosen time allowed for assessing the taste preferences during a specific growth stage of the broiler chickens. The test was carried out during the initial stage of development, where they exhibit more pronounced preferences [26]. Two sapid compounds were evaluated, delivered via a water matrix: sucrose (representing sweet taste) at 100 mM and monosodium glutamate (MSG, representing umami taste) at 300 mM (Prinal S.A., Santiago, Chile). During each test, the broilers had simultaneous access to two identical drinkers, namely one containing pure water and the other containing the test solution (either sucrose or MSG) positioned 20 cm apart, following the methodology developed by our group and described in Cordero et al. [26]. The preference tests lasted 2 h, starting at 10 a.m. and ending at noon, with a one-hour fasting period starting at 9 a.m. Access to the ad libitum drinkers within each pen was restricted during this period. During the four days of preference testing, sucrose and MSG were tested in a counterbalanced manner across both treatments. Specifically, half of the pens in each treatment began with sucrose presented on one side and then on the other side, followed by MSG presented on the left and on the right. This counterbalancing was implemented to avoid the potential biases and ensure the reliability of the preference data. The average consumption from each drinker was estimated by the weight loss of the matrices, calculated by subtracting the amount supplied from the amount removed 2 h after the start of the test. The consumption of the birds was adjusted based on their metabolic weight for each test day and expressed in grams per kilogram (g/kg) of body weight (BW). The preference value was calculated as the percentage consumption of sucrose or MSG concerning total intake (consumption of sucrose or MSG plus water consumption) and compared to the neutral value of 50%, as described in Cordero et al. [26].

### 2.6. Tonic Immobility Test

To induce tonic immobility on days 20 and 40, methods based on Wang et al. [36] were used for all the chickens. The researcher selected each chicken manually and carried it to an adjacent room. Both researchers wore the same attire: dark green coveralls and black boots. Each bird was placed on its back on a white metal table, measuring 2 m in length and 50 cm in width. The bird was immobilized for 15 s, with one hand on the sternum and the other holding its head. A second researcher was responsible for starting the stopwatch after the 15 s of immobilization, when the first researcher released the bird silently, remaining quiet and nearly motionless. The first researcher, responsible for handling the birds during tonic immobility, was different from the one who performed the gentle handling interactions, whereas the second researcher, who timed the procedure, was the same as the one responsible for the gentle handling. If the bird righted itself in less than 10 s, tonic immobility was considered not induced, so the procedure was attempted again, with a maximum of 3 attempts per bird. If more than 10 s passed before the bird righted itself, tonic immobility was considered induced, and the induction time was recorded for up to 10 min, after which the test was concluded.

### 2.7. Attention Bias Test

To determine the affective state of the broiler chickens, at the end of the experimental period (day 43), an attention bias test was conducted for 64 birds (4 birds per pen; 32 birds per treatment) based on a study by Anderson et al. [27]. The birds were evaluated in an experimental pen of 2 m^2^, located in a separate room adjacent to the experimental unit. The birds were randomly selected from their pen. Prior to testing, feed was restricted for 20 min to increase their motivation to eat. The arena had the same substrate as the rearing pens, consisting of wood shavings, and the walls were made of metal, identical to the ones used in the rearing pens, ensuring a familiar environment for the birds.

The test arena was recorded using IP Wi-Fi 2MP cameras (Ezviz, SENKO INGENIERIA SPA, Santiago, Chile), positioned at a height of 50 cm above the pens and securely mounted to the top of the pen structure, attached to the fencing. This setup ensured a stable and unobstructed view of the experimental arena. The cameras were connected and monitored via Wi-Fi on a computer using software from the same brand. Additionally, the setup ensured that the chickens not being tested could not hear or see the ones in the experimental pen, minimizing any external influences on their behavior during the evaluation.

To reduce the stress due to isolation, the birds were tested in groups of four from the same pen, based on the findings from Jones [37] who demonstrated the beneficial effect of social presence in reducing the stress responses in domestic chicks. The arena was cleaned between the groups, with the wood shavings replaced and the food replenished, if necessary, to ensure the consistency of the testing conditions.

The test lasted a maximum of 5 min. The birds were transported together in a box to the experimental unit, and after a waiting period of approximately 2 min, all four birds were simultaneously placed in the center of the arena. No habituation was provided for the birds prior to either the transport or the experimental arena. The test began when the birds were placed in the center of the arena, where there was a small pile of their usual commercial feed (500 g). Once in the experimental pen, a specific 5 s aversive sound consisting of dog barking was played from a portable speaker (Harman Kardon Onyx 6, Harman Kardon, Santiago, Chile). The latency to start feeding after the aversive sound was recorded using video. If the bird resumed feeding from the feed pile, the aversive sound was played a second time after 5 s to assess if it was sufficiently threatening to interrupt feeding and to measure the bird’s readiness to resume eating. The test ended when the bird resumed feeding for the second time, or after 5 min had elapsed. In addition to the latency to resume feeding, video recordings were used to measure the latency to the first step, time spent feeding, and latency to finish feeding. Vigilance behaviors (erect posture, neck stretching, looking around, freezing) were also observed following the first aversive sound and coded using BORIS software 7.13.9 [33]. Each vigilance behavior was scored as 0 (not observed) or 1 (observed), resulting in a vigilance score between 0 (no vigilance behaviors observed) and 4 (all vigilance behaviors observed). Recording the latency to vocalize was not feasible due to the group testing.

### 2.8. Growth Performance

All the broilers were weighed by pen on days 7, 14, 21, 28, 35, and 42 to assess their body weight (BW) and feed intake during these periods. These measurements were used to calculate the average daily gain (ADG) and average daily feed intake (ADFI). The feed conversion ratio (FCR) was determined by dividing the ADG by the ADFI. The mortality was recorded throughout the experimental period, and any deceased birds were accounted for in the feed intake adjustments to ensure accurate FCR calculations.

### 2.9. Statistical Analysis

The analyses were conducted using RStudio 4.1.0. [38]. All the data were checked for homoscedasticity using Levene’s test and for normality using the Shapiro-Wilk test. A logarithmic transformation was applied to the time variable in the tonic immobility test to meet the assumptions of normality and homoscedasticity. The significance level (α) was set at 0.05. However, the results with *p*-values between 0.05 and 0.10 were considered to indicate trends, which suggest the potential effects that approach significance. This practice allows for the identification of patterns that may not meet the strict significance criteria but still warrant consideration for further investigation. For the behavioral analyses, a mixed repeated-measures ANOVA was used to analyze the time spent feeding, drinking, locomotion, lying, and standing behaviors. In this model, the treatment, time, and treatment × time interaction were included as fixed effects, and the pen was treated as a random effect. Binomial logistic regression was employed to assess the occurrences of preening, foraging, mock fighting, dustbathing, and the use of enrichments, with behaviors scored on a scale of 0–1, indicating the presence or absence of these specific behaviors. Footpad lesions were evaluated qualitatively, and no statistical tests were necessary due to the absence of lesions across both treatments. For the sweet and umami taste preference analysis, a *t*-test was used to compare the preferences for sweet and umami substances relative to the neutral value. For the tonic immobility test, the latencies were compared between the treatments using a two-sample *t*-test. In the attention bias test, general linear mixed effects models (GLMMs) were used to analyze the latencies to first step, begin feeding, time spent feeding, and resume feeding. In these models, the treatment was considered a fixed effect, while the ID animal was treated as a random effect to account for the individual variability among the animals. Vigilance behavior scores (erect posture, neck stretching, looking around, freezing) were also assessed using GLMMs. Treatment was considered a fixed effect, while the pen was treated as a random effect.

Finally, for growth performance, a one-way ANOVA was performed to analyze the ADG, ADFI, and FCR. The mortality was recorded throughout the experimental period, and deceased birds were accounted for in the feed intake adjustments to ensure accurate FCR calculations.

## 3. Results

### 3.1. Behavioral Analysis

**Figure 4:**
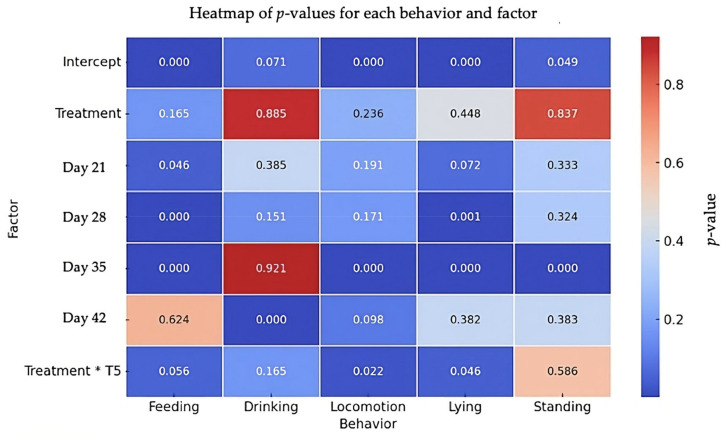
Heatmap of *p*-values for feeding, drinking, locomotion, lying, and standing behaviors. The heatmap shows *p*-values for each behavior (feeding, drinking, locomotion, lying, standing). Treatment refers to the effect of the Social group (positive human–animal interactions) with the Control group (physical enrichment, including perches, straw bales, and platforms) as the reference. Treatment * T5 refers to interaction of treatment and day 42. Colors indicate the significance of the effects, with red representing less significant effects (higher *p*-values) and blue indicating more significant effects (lower *p*-values).

The results of the behavioral analysis are summarized in Figure 4. No significant overall effect of the social treatment was observed in any of the behaviors analyzed. However, when examining specific time points, some significant differences were found. For feeding behavior, the feeding time increased significantly at day 21 (β = 36.12, SE = 18.06, *t* = 2.00, *p* = 0.046) and decreased significantly at day 28 (β = −66.58, SE = 18.06, *t* = −3.69, *p* < 0.001) and day 35 (β = −74.93, SE = 18.06, *t* = −4.15, *p* < 0.001), with a marginal interaction effect observed at day 42 (β = 49.29, SE = 25.82, *t* = 1.91, *p* = 0.056). For drinking behavior, while there was no significant effect of the social treatment (β = 0.86, SE = 5.90, *t* = 0.15, *p* = 0.885), a significant increase in drinking time was noted at day 42 (β = 20.70, SE = 5.43, *t* = 3.81, *p* < 0.001). Regarding locomotion behavior, no significant treatment effect was found (β = 19.28, SE = 16.10, *t* = 1.20, *p* = 0.236). However, locomotion was significantly reduced at day 35 (β = −98.43, SE = 18.43, *t* = −5.34, *p* < 0.001), and a significant interaction effect between the social treatment and time was detected at day 42, indicating a reduction in locomotion under the social treatment (β = −67.60, SE = 29.49, *t* = −2.29, *p* = 0.022). For lying behavior, there was no overall effect of the social treatment (β = 24.65, SE = 32.32, *t* = 0.76, *p* = 0.448), but significant decreases in lying time were observed at day 28 (β = −73.03, SE = 21.87, *t* = −3.34, *p* = 0.001) and day 35 (β = −107.80, SE = 21.87, *t* = −4.93, *p* < 0.001). A significant interaction effect at day 42 showed an increase in lying time under the social treatment (β = 91.56, SE = 45.82, *t* = 1.99, *p* = 0.046). Finally, for standing behavior, no significant effect of the treatment was observed (β = −1.14, SE = 5.55, *t* = −0.21, *p* = 0.837), although a significant increase in standing time was noted at day 35 (β = 21.20, SE = 5.16, *t* = 4.11, *p* < 0.001).

Due to the low occurrence of mock fighting (3.7%), allopreening (1.7%), dustbathing (1%), and use of enrichment (3.5%), these behaviors were excluded from the logistic regression analysis. The enrichment behavior was only used in the control treatment, which further justified its exclusion from the analysis. Therefore, the focus was placed on preening (20.4% occurrence) and foraging (17.4% occurrence), which showed higher frequencies of occurrence. Through logistic regression, no significant effect of the social treatment was found for preening (*p* = 0.233, OR = 0.7358, 95% CI [0.445, 1.218]), indicating that the treatment did not influence this behavior. However, for foraging, the social treatment had a significant positive effect (*p* = 0.027, OR = 3.761, 95% CI [1.170, 12.091]), suggesting that birds receiving the social treatment were more likely to engage in foraging compared to those receiving the control treatment (Table 3).

### 3.2. Footpad Lesions

Throughout the assessment period, none of the chickens exhibited adverse conditions; specifically, there were no observed injuries on the feet of any of the subjects from both the control and social treatments.

### 3.3. Sweet and Umami Taste Preference Analysis

**Figure 5:**
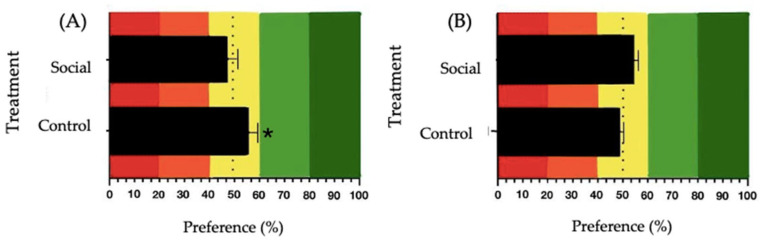
Preference values for: (**A**) sucrose 100 mM and (**B**) MSG 300 mM solutions in broiler chickens. The control treatment involved physical enrichment (perches, straw bales, and platforms), while the social treatment involved positive human-animal interactions. Red and orange colors in the graphs represent the aversion zone, yellow color the indifference zone and green the preference zone. The dashed line indicates the 50% preference threshold. The asterisk (*) denotes significant differences (* *p* ≤ 0.050).

For sucrose 100 mM, a significant preference was observed in the Control group (*t*(6) = 3.060, *p* = 0.022, mean = 56.90, SE = 2.25), while no significant preference was observed in the Social group (*t*(7) = −0.104, *p* = 0.920, mean = 49.74, SE = 2.53) (Figure 5A). For MSG 300 mM, no significant preference was observed under either treatment. In the Control group, the analysis resulted in *t*(7) = −0.843, *p* = 0.427 (mean = 48.03, SE = 2.34), and for the Social group, the results were *t*(7) = 0.540, *p* = 0.606 (mean = 52.01, SE = 3.73) (Figure 5B).

### 3.4. Tonic Immobility Test

**Figure 6:**
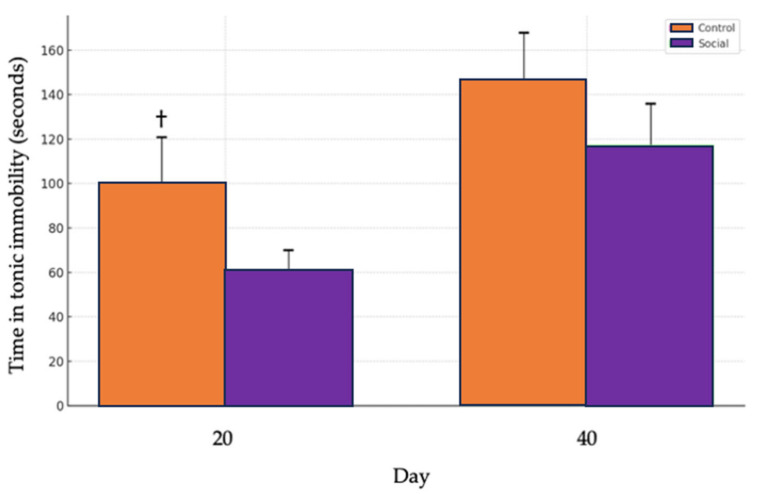
Comparison of tonic immobility times between control and social treatments. The Control group is represented by the orange bars, while the Social group is shown with purple bars. The bars indicate the average time spent in tonic immobility on two different days, day 20 and day 40. The error bars represent the standard error of the mean (SEM) for each treatment, illustrating the variability within the data. (†) denotes a trend (*p* = 0.078) toward significance.

A tendency for higher tonic immobility times was observed in the Control group (physical enrichment) compared to the Social group (social enrichment) on day 20 (t = 1.4281, df = 90.675, *p* = 0.078), indicating a trend toward significance. No significant differences between treatments were observed on day 40 (t = 0.83629, df = 88.415, *p* = 0.405) (Figure 6).

### 3.5. Attention Bias Test

The results of the GLMMs (Table 4) show that for the first step model, the intercept is 10,221.13 (*p* = 0.05), indicating that the average latency for the first step is significantly different from zero. The treatment effect (Social group vs. Control group) was not significant (coefficient: −999.13, *p* = 0.776), and the pen does not significantly affect the latency to take the first step (coefficient: 148.58, *p* = 0.269). For the Begin Feeding model, the intercept is 22,749.20 (*p* < 0.001), suggesting that the broilers, on average, began feeding after this time. The treatment effect is significant (coefficient: −8830.44, *p* = 0.006), indicating that the broilers in the Social group started feeding earlier than those in the Control group. The pen had a marginally significant effect (coefficient: 952.77, *p* = 0.059), suggesting that the pens may slightly influence feeding behavior. In the Time Spent Feeding model, the intercept is 38,278.47 (*p* < 0.001), meaning that the broilers, on average, spent this amount of time feeding. The treatment effect is not significant (coefficient: −7510.47, *p* = 0.108), but the pen had a significant effect (coefficient: 599.32, *p* = 0.027), indicating that the pen location affected how long the broilers spent feeding. For the Resume Feeding model, the intercept is 38,278.47 (*p* < 0.001), indicating that the broilers finished feeding after this time on average. The treatment effect is not significant (coefficient: −7510.47, *p* = 0.108), suggesting no statistically significant difference between the treatments in the time taken to finish feeding. However, the pen had a significant effect (coefficient: 599.32, *p* = 0.027), indicating that the broilers in different pens took varying times to finish feeding, possibly due to the differences in the pen conditions.

Table 5 shows that significant differences were observed only for the freezing behavior. The social treatment significantly increased the likelihood of chickens exhibiting this behavior compared to the Control group (coefficient = 4.556, SEM = ±1.867, *p* = 0.015). Additionally, the pen, considered as a random effect, also had a significant influence on the occurrence of freezing (coefficient = −1.053, SEM = ±0.403, *p* = 0.009). Regarding erect posture, no significant differences were found between the treatments (coefficient = 0.880, SEM = ±0.752, *p* = 0.194); however, the effect of the pen was significant (coefficient = −0.220, SEM = ±0.080, *p* = 0.006). For neck stretching, no significant differences were observed between the treatments (coefficient = −0.577, SEM = ±0.555, *p* = 0.443), nor was the effect of the pen significant (coefficient = 0.088, SEM = ±0.083, *p* = 0.287). Similarly, looking around showed no significant differences between the treatments (coefficient = −0.577, SEM = ±0.752, *p* = 0.443), and the effect of the pen was also not significant (coefficient = 0.088, SEM = ±0.083, *p* = 0.287).

### 3.6. Growth Performance

The growth performance of the broiler chickens was evaluated over a 42-day period. During the first week, a total of four birds died: three from the social enrichment treatment and one from the physical enrichment treatment. These mortalities occurred during the critical early stage, which is often associated with increased vulnerability to environmental- and management-related factors. These isolated cases were within the expected range for commercial broiler production systems. No significant differences were observed between the control and social treatments across any of the growth parameters. Specifically, the initial BW did not differ significantly between the control and social treatments (F(1,14) = 0.39, *p* = 0.5417). The ADFI was similar between the treatments, with no significant difference (F(1,14) = 0.02, *p* = 0.9240). The ADG also showed no significant differences (F(1,14) = 0.01, *p* = 0.9408), and the FCR was not significantly different between the treatments (F(1,14) = 0.23, *p* = 0.6420). Finally, no significant differences were found in the final BW between the treatments (F(1,14) = 0.01, *p* = 0.9383) (Table 6).

## 4. Discussion

The present study compared two different enrichment strategies for broilers: a control treatment with physical enrichment (perches, platforms, and straw bales) and a social treatment that involved positive human-animal interactions. These strategies were used to assess their effects on broiler welfare, including behavioral outcomes, footpad health, taste preferences, affective states, and growth performance. It was hypothesized that broiler chickens exposed to regular positive human interactions (social enrichment) will demonstrate an increased expression of exploratory behaviors, such as locomotion and foraging activity, reduced footpad lesions, a preference for sweet and umami taste compounds, and a more positive affective state compared to birds that receive only physical enrichment without any human interaction.

No significant overall effect of the social treatment was observed in any of the behaviors analyzed. However, when examining specific time points, some significant differences were found. When examining the interaction between the treatments and the end of the experimental period (day 42), the broilers receiving the social treatment exhibited reduced locomotion and increased lying behavior compared to the Control group. This suggests that by the end of the experimental period, the positive human-animal interactions influenced these behaviors, possibly leading to greater resting behavior. While this increase in lying time could reflect a more restful state or comfort, the reduction in locomotion raises concerns regarding the potential welfare implications, such as increased risk of contact dermatitis from prolonged lying [1]. This finding contrasts with the general aim of enrichment strategies, which is to promote more movement and exploratory behaviors to enhance broiler welfare [6]. The locomotion behavior and foraging behavior in this study appeared to show opposite trends, which can be explained by the way that each behavior was measured. Locomotion was recorded when the birds moved from one location to another, walking or running, without engaging in any other activity. In contrast, foraging was specifically recorded when the birds were scratching at the ground, often accompanied by one or two steps backward after scratching. If the birds were moving while performing these actions, it was categorized as foraging rather than locomotion. Consequently, if we had considered foraging within the locomotion category, it is likely that the total time spent in locomotion would have shown a significant increase, reflecting the birds’ movement while engaging in foraging activities. This suggests that positive human–chicken interaction promotes more natural behaviors, related to foraging. These findings are consistent with previous studies demonstrating that regular human contact encourage exploratory behaviors in broilers [27,39].

No footpad lesions were observed in either treatment, indicating that the environmental conditions were conducive to maintaining physical health in both treatments. Footpad lesions are commonly associated with poor litter quality and high moisture levels, but the absence of such lesions in this study reinforces that enrichment strategies, are crucial for maintaining the physical health of broilers, regardless of the enrichment type. This finding aligns with research suggesting that platforms and straw bales can mitigate issues like pododermatitis and walking difficulties in high-density environments, which highlights the role of physical enrichments in preventing health-related welfare issues [21]. However, while no lesions were observed in either treatment, we cannot conclusively attribute the absence of pododermatitis to either physical or social enrichment.

The analysis of sweet and umami taste preferences revealed that the control treatment birds showed a significant preference for sucrose, while the social treatment birds did not. This could be explained by the differences in the birds’ affective states, which refer to their emotional experiences, which can range from short-term reactions like fear to more prolonged emotional conditions such as anxiety or comfort. These states are critical for understanding animal welfare, as they directly influence behaviors and preferences. Forbes [25] suggested that animals might seek specific taste compounds, to alleviate the negative affective states caused by stress or discomfort. In stressful environments, animals may display heightened preferences for hedonic stimuli, like sucrose or monosodium glutamate (MSG), as a coping mechanism to improve their emotional welfare. The differences in sweet and umami taste preferences between the control and social treatments could be attributed to variations in the birds’ affective states. The birds receiving the control treatment, which only received physical enrichment, may have experienced more stress or discomfort, potentially driving their preference for sucrose as a means to enhance their emotional state. In contrast, the birds receiving the social treatment, who benefited from positive human interactions, may have experienced a more positive affective state, reducing the need for external rewarding stimuli, such as sucrose, to achieve comfort. This finding aligns with [39] which suggested that socially enriched birds may experience a more fulfilling environment, thus having less need for external hedonistic stimuli such as sucrose [40]. In contrast, the birds in the Control group may have relied on sucrose as a means to improve their emotional state, reflecting their preference for this compound. The absence of a preference for MSG in both groups was unexpected, as previous research from our group showed that broilers prefer this umami compound at this concentration [26] and raises questions about the relationship between taste preference and environmental enrichment. It is possible that individual variability, developmental changes, or subtle differences in the experimental conditions, as noted by Taylor et al. [41], could have influenced the outcomes. The inclusion of the taste preference test introduces a new dimension to welfare assessment by linking emotional states to taste preferences. The findings related to MSG are particularly intriguing and warrant further investigation. Exploring a broader range of taste stimuli could provide more comprehensive insights into the birds’ emotional states and help to clarify how environmental enrichment strategies, such as social interaction, modulate these responses over time. Understanding this relationship would offer deeper insights into animal welfare and the complex interplay between affective states, behavior, and taste preferences.

The tonic immobility test revealed a trend where the control treatment exhibited longer immobility times on day 20, suggesting a potential increase in fear levels, although this difference was not statistically significant (*p* = 0.078). Tonic immobility is often used as a measure of fearfulness, with longer durations indicating heightened fear responses [36]. While the trend observed aligns with our hypothesis that physical enrichment alone may not be sufficient to reduce fear, it is important to note that the difference did not reach statistical significance. Additionally, unlike the findings reported in other studies, we found no significant differences between treatments on day 40 (*p* = 0.405). This suggests that the potential effects of social enrichment on fear behavior may diminish over time or become less detectable at later stages in the birds’ development. The absence of significant differences on day 40 emphasizes the dynamic nature of behavioral responses and affective states, which can fluctuate throughout the production cycle. This result highlights the importance of considering the timing of behavioral assessments, as well as the possibility that other factors, such as habituation to the environment or changes in social dynamics, may have influenced the birds’ responses by this stage. Our decision to report the trend is discussed to encourage a further exploration rather than to assert definitive conclusions. This approach allows for the identification of patterns that may not meet the strict significance criteria but still provide meaningful biological insights, particularly in behavioral studies where variability is common. The trend toward reduced immobility times receiving the social treatment suggests that regular human interaction may have had a calming effect, potentially lowering the birds’ fear and stress levels. This observation aligns with the findings from other studies demonstrating the positive effects of human contact on reducing the fear responses in broilers [39]. However, given the lack of statistical significance, these results should be interpreted with caution and explored further in future research with larger sample sizes or extended exposure to social enrichment. It is also relevant to note that the tonic immobility test was conducted by a different individual than the one who provided the gentle handling, though both individuals wore the same dark green coveralls. This consistency in clothing could have contributed to a more familiar environment, potentially influencing the birds’ responses. Future research could explore these variables further to better understand their impact on behavior and fear responses throughout the production cycle.

The attention bias test further supported the benefits of social enrichment, as the birds receiving the social treatment were quicker to begin feeding after an aversive sound (an audio recording of dog barks) compared to those receiving the Control treatment. This suggests that the socially enriched birds were less vigilant and more confident in resuming normal activities, indicating lower anxiety levels and potentially a more positive affective state [27,28]. However, the unexpected increase in freezing behavior under the social treatment challenges the assumption that social enrichment universally reduces vigilance, suggesting complexities in the emotional responses of the birds that align with the findings reported by Edgar et al. [42], who demonstrated that while social interactions can reduce certain anxiety-related behaviors, they may also heighten the sensitivity to sudden or unfamiliar stimuli under specific conditions. This suggests that freezing, as a behavioral response, may not solely indicate heightened fear but rather reflect a context-specific form of vigilance or coping strategy. While social enrichment is generally associated with improved welfare through reduced anxiety and increased positive affect, this result highlights that its effects may not be uniform across all fear responses. The increase in freezing behavior may reflect heightened situational vigilance or a context-specific coping strategy that emerges under certain conditions, despite the overall reduction in anxiety levels. These findings suggest that social enrichment may simultaneously promote both positive and heightened emotional responses, depending on the context and type of stimulus encountered. It is possible that while social interactions help birds feel safer in their environment, they may also increase their sensitivity to unfamiliar threats, leading to increased freezing behavior. Future studies should investigate these dual effects of social enrichment to better understand how specific social interactions influence both anxiety reduction and vigilance. Exploring these dynamics in more detail will provide deeper insights into the conditions under which social enrichment promotes positive welfare outcomes, while also identifying situations where it may enhance certain fear behaviors.

The lack of significant differences in growth performance between the control and social treatments indicates that social enrichment can be implemented, maintaining the growth outcomes. The similar ADG, FCR, and final BW between both treatments confirm that welfare improvements achieved through social enrichment can be implemented, maintaining growth outcomes. This finding is in line with other research showing that welfare enhancements can coexist with sustained production efficiency [40,43] which is an encouraging outcome for both animal welfare and economic sustainability.

The results of this study contribute to the growing body of evidence supporting the use of enrichment, particularly social enrichment, to enhance broiler welfare. While physical enrichment is widely used, this study highlights the potential benefits of incorporating human-animal interactions into management practices. Social enrichment not only improved the behavioral outcomes, such as increased foraging, but also demonstrated its potential to positively influence the affective state of broilers, as seen in their lower levels of fear and quicker recovery after stressful events. Moreover, it maintained physical health and productivity, making it a viable option for producers aiming to enhance both animal welfare and production sustainability.

Future research should explore the progressive impact of social enrichment on broilers, particularly in terms of its impact on fear behavior and overall welfare. Additionally, the unexpected results regarding freezing behavior and the lack of significant preferences for the umami taste suggest that more research is needed to fully understand how social interactions affect broiler cognition and taste perception. Studies that examine the interaction between different types of enrichment (e.g., combining physical and social) could also provide valuable insights into optimizing broiler welfare in intensive systems. Finally, investigating the economic viability of implementing social enrichment on a larger scale could help facilitate its adoption in commercial settings.

## 5. Conclusions

Social enrichment through positive human–animal interactions promotes foraging behavior, and seemed to promote a more positive affective state in the broilers, contributing to their overall welfare. This enrichment strategy maintains physical health, and growth performance, highlighting its potential for improving animal welfare in intensive production systems alongside sustained productivity. The study emphasizes the importance of considering both physical and social enrichment in broiler management. The inclusion of positive human–animal interactions not only supports behavioral welfare improvements but also enhances the birds’ affective state, offering a promising approach to fostering more humane and sustainable poultry production systems while maintaining productivity.

## Figures and Tables

**Table 1:** Adaptation of ethogram used for exploration and continuous observation of *Gallus gallus domesticus* from Sanchez-Casanova et al. [34].

Category	Behavior *	Description
Individual	Feeding	Eating from a food hopper, whilst standing, sitting, or resting.
	Drinking	Drinking from the water trough, whilst standing, sitting, or resting.
	Locomotion	Moving by walking or running.
	Lying	Main part of the body touching the ground, either chest or side.
	Standing	The abdomen not touching the litter or ground, and the bird is motionless with no apparent movement of the legs.
	Preening	Moving beak along the plumage.
Interaction with the environment	Dustbathing	While lying with fluffed feathers, the bird simultaneously and rapidly lifts its wings up and down multiple times, while scooping loose substrate material up into the feathers.
	Use of enrichments	Interaction with environmental enrichments such as perches, platforms, or straw bales, including climbing, perching, or pecking at the enrichments.
	Foraging	Scratching at the ground, with intermittent bouts of ground pecking items (visible or not). Usually followed by one or two steps backwards after a bout of ground scratching.
Social Interaction	Mock fighting	After running toward each other, birds stop and stare at each other. The interaction is brief, harmless to the birds, and not directed persistently at any one bird.
	Allopreening	The birds use their beaks to arrange the feathers of another bird.

* The behaviors are categorized into three main types: individual, which includes actions such as feeding, drinking, locomotion, lying, standing, and preening; interaction with the environment, which involves behaviors like dustbathing, use of enrichments and foraging; and social interaction, where birds engage with one another in activities such as mock fighting and allopreening.

**Table 2:** Description of podal fitness scores as derived from Welfare Quality [35].

Fitness Assessment	Score *	Descriptions
Footpad condition	0	Feet intact, no or minimal proliferation of the epithelium.
	1	Necrosis or proliferation of the epithelium or chronic bumble foot with no or moderate swelling.
	2	Swollen (dorsally visible).
	3	Moderate footpad dermatitis.
	4	Severe footpad dermatitis.

* The data reflect the classification criteria for footpad condition severity in the broiler chickens, providing a standardized assessment for welfare evaluation. The score ranges from 0 to 4, with higher scores indicating greater severity.

**Table 3:** Occurrence and binomial logistic regression analysis of preening and foraging behaviors.

Behaviors	Occurrence (%)	*p*-Value (Regression)
Mock fighting	3.7%	-
Allopreening	1.7%	-
Dustbathing	1%	-
Enrichment Use	3.5%	-
Preening	20.4%	0.233
Foraging	17.4%	0.027 *

* Indicates a statistically significant result (*p* ≤ 0.050), while the - symbol represents behaviors that were excluded from the analysis due to their low occurrence percentages (below 4%) and therefore have no associated *p*-value.

**Table 4:** Latency to first step, begin feeding, time spent feeding, and resume feeding.

Latencies (s)	Intercept (*p*-Value)	Treatment (*p*-Value)	Pen (*p*-Value)
First step	0.05 *	0.776	0.269
Begin feeding	<0.001 *	0.006 *	0.059
Time spent feeding	<0.001 *	0.108	0.027 *
Resume feeding	<0.001 *	0.108	0.027 *

* Indicates statistical significance (*p* ≤ 0.050). The values represent the *p*-values for the intercept, treatment (Social vs. Control), and pen effects on the latencies for first step, begin feeding, time spent feeding, and resume feeding, analyzed using a General Linear Mixed Model.

**Table 5:** Coefficient ± SEM for vigilance behavior scores in the attention bias test.

Behaviors	Social Treatment	Control Treatment	Pen (Random Effect)
Erect posture	−0.577 ± 0.752	Ref	−0.220 ± 0.080 * (*p* = 0.006)
Neck stretching	−0.029 ± 0.555	Ref	0.088 ± 0.083
Looking around	−0.577 ± 0.752	Ref	0.088 ± 0.083
Freezing	4.556 ± 1.867 * (*p* = 0.015)	Ref	−1.053 ± 0.403 * (*p* = 0.009)

This table summarizes the coefficients (± SEM) for four behaviors in the broiler chickens under two treatments: Control (physical enrichment: perches, straw bales, and platforms) and Social (social enrichment: positive human-animal interactions). The control treatment is used as the reference (Ref), and the coefficients for the social treatment indicate how the likelihood of each behavior changes compared to the reference. Positive coefficients suggest an increase in behavior likelihood under the social treatment, while negative coefficients indicate a decrease. The Pen (Random Effect) shows variability between the pens where the chickens were housed, and statistically significant *p*-values (*p* ≤ 0.050) are marked with an asterisk (*). SEM reflects the variability in the coefficient estimates.

**Table 6:** Productive parameters of birds during a 42-day production cycle.

Days 1 to 42	Control	Social	SEM ^5^	*p*-Value
	(*n* = 48)	(*n* = 48)		
Initial BW ^1^ (g)	41.0	40.7	0.396	0.542
ADFI ^2^ (g)	92.4	92.5	0.728	0.924
ADG ^3^ (g)	49.9	50.0	1.169	0.941
FCR ^4^	0.5	0.6	0.019	0.642
Final BW (g)	2136.6	2142.1	49.041	0.938

^1^ (BW) Body weight measured in grams. ^2^ (ADFI) Average daily feed intake of a 42-day production cycle, measured in grams per day. ^3^ (ADG) Average daily gain of a 42-day production cycle, measured in grams per day. ^4^ (FCR) Feed conversion ratio of a 42-day production cycle, determined by dividing the ADG by the ADFI. ^5^ (SEM) standard error of the mean. The control treatment involved physical enrichment (perches, straw bales, and platforms), while the social treatment involved positive human-animal interactions.

## Data Availability

The data supporting the findings of our study is openly available on the Mendeley Data repository at the following link: https://data.mendeley.com/datasets/g58gz323xv/1. DOI: 10.17632/g58gz323xv.1.
